# Surveying the arable plant diversity of conventionally managed farmland: a comparison of methods

**DOI:** 10.1007/s10661-019-8042-7

**Published:** 2020-01-07

**Authors:** Alexander Wietzke, Christoph Leuschner

**Affiliations:** Department of Plant Ecology and Ecosystems Research, University of Goettingen, Untere Karspüle 2, 37073 Goettingen, Germany

**Keywords:** Alpha diversity, Arable weeds, Field edge, Field interior, Plant detection rate, Plot size

## Abstract

Agricultural intensification has led to drastic population declines in Europe’s arable plant vegetation, and continuous monitoring is a prerequisite for assessing measures to increase and conserve remnant populations of endangered arable plant species. Unfortunately, strong variation in plot sizes and in-field locations makes comparison of current arable plant monitoring approaches difficult. This study compares different relevé approaches in conventionally managed arable fields in Northwest German farmland with respect to plant species detection success and time expenditure. We compared species detection rate and expenditure of time of six different relevé types in 45 conventionally managed arable fields (each 15 fields of wheat, maize, and rapeseed): field “Interior” plots (50 × 2 m); field edge plots: “Edge_30” (30 × 2 m), “Edge_50” (50 × 2 m), and “Edge_500” (500 × 1 m); “Subplots” (four dispersed plots of 5 × 1 m); and “Corner” plots (50 × 2 m). To determine species detection rate, the species richness recorded with a survey method was related to the field’s total plant species number as estimated from a survey of the entire field edge zone. With a species detection rate of 8.3% (median), interior plots were inadequate for characterizing the field’s arable plant vegetation. Edge_500 plots yielded the highest proportion of the field’s arable plant species pool (75.6%, including taxa of conservation value), followed by “Corner” plots (45.8%) and “Sublots” (32.6%). Edge_50 and Edge_30 plots detected less than 25% of the field’s species pool. The average time needed for a relevé was 20 min in Edge500 plots and 5–11 min in the other plot types. We suggest implementing Edge_500 plots as a standard monitoring approach in conventionally managed farmland due to its favorable ratio of detection success to expenditure of time. Our findings should be compared to methodological studies conducted in other regions, in different farmland management systems, and in landscapes of variable complexities.

**Electronic supplementary material:**

The online version of this article (10.1007/s10661-019-8042-7) contains supplementary material, which is available to authorized users.

## Introduction

In former times, crop cultivation in the farmland was less intense, and many plant species were able to coexist with the crop. In Central Europe, about 300–350 plant species have adapted to the frequently disturbed man-made habitat of arable fields. These weed species formed characteristic arable plant communities that have accompanied agriculture since centuries, if not millennia (Leuschner and Ellenberg 2017). Since the 1950s–1960s, advanced soil cultivation techniques, the widespread application of herbicides, and increased fertilizer amounts that intensified competition with the crop have caused dramatic impoverishment of the arable plant vegetation in many regions of Central Europe and elsewhere, which manifested in large losses of arable plant cover and species richness and the collapse of once widespread arable plant (segetal) communities (Albrecht et al. 2016; Albrecht 1989; Májeková et al. 2010; Meisel and von Hübschmann 1976; Meyer et al. 2013). From a literature review, Leuschner and Ellenberg (2017) concluded that, in comparison to other habitats in Central Europe, the species richness and population size of arable plants have experienced the most significant decrease within the past 50–60 years. For example, the study of Kläge (1999) in south-eastern Germany in the 1990s found that of 282 formerly recorded segetal species in the region, 90 taxa have disappeared and 72 showed a frequency decline of different extent. Albrecht (1989) observed a decline in the arable plant species pool of 20% between the intervals 1951–1968 and 1968–1988 in Bavaria, which was associated with a reduction in plot-level species richness from 23 to 16 species on average. In a large number of fields of Central and Northern Germany, the resampling study of Meyer et al. (2013) found a 23% reduction in the regional species pool and a decrease in median plot-level species richness from 24 to 7 species in the field interior compared to the 1950s–1960s. The species loss was associated with a large decline in arable plant coverage. Meyer et al. (2013) found that the median cover of arable plants declined from 30% in the 1959s–1960s to only 3% in 2009 in the interior of central and northern German fields.

Arable plants (“weeds”) have always been a bane of the farmers due to reduced crop yield, but they are also fulfilling important ecosystem functions in the farmland, the benefits of which have only been fully recognized in the recent past. The root system of a sufficiently dense cover of arable plants can reduce soil erosion, and the plants may catch nutrients (in particular mobile nitrate) during the summer fallow, thereby reducing leaching loss (Gholamhoseini et al. 2013). Equally important are positive effects on pollinator communities and related pollination success of crops and pest control through food webs supported by arable plants (Hawes et al. 2003; Hyvönen and Huusela-Veistola 2008; Médiène et al. 2011; Wietzke et al. 2018). Arable plants also support herbivorous insects which are eaten by insectivorous birds, and they provide food for granivorous birds (Marshall et al. 2003). In Northern Germany, Heydemann and Meyer (1983) counted 1200 insect species colonizing 102 arable plant species.

Thus, arable plants are of particular interest for biodiversity conservation in agricultural landscapes, and various agri-environmental schemes such as conservation headlands have been introduced to halt plant biodiversity loss and reverse the trend in Europe (European Union 2013a, b, 2014a, b).

To assess the status quo of the segetal flora, monitor long-term trends, and evaluate the effects of environmental measures in the farmland, a consistent monitoring concept is needed. Vegetation surveys in the farmland have used a variety of plot sizes and plot geometries in the past, with plot size varying between 0.1 and 5000 m^2^ (Chytrý and Otýpková 2003; Hanzlik and Gerowitt 2016; Lososová et al. 2004; Meyer et al. 2013; Richner et al. 2015). Based on a comprehensive European dataset considering 2604 arable plant relevés between 1970 and 2000, Chytrý and Otýpková (2003) found a mean plot size of 74 m^2^, whereas the most frequently used plot size was 10 m^2^. For surveying arable plants, vegetation ecologists have traditionally made relevés in square or rectangular plots of 25 to 100 m^2^ size (Dierschke 1994; Hanzlik and Gerowitt 2016). For the analysis of the rich arable plant vegetation of the 1950s to 1970s, plots of 25 m^2^ were generally sufficiently large; this is certainly no longer possible in the nowadays intensively managed, species-poor farmland. In recent time, authors have investigated plots of largely different size, form, and location in the field, when investigating different aspects of the arable plant vegetation. Examples of single-plot approaches are 100-m^2^ plots in the field interior (Fahrig et al. 2015; Lüscher et al. 2014) and 60-m^2^ plots at least 3 m distant to the field edge (German Federal Agency for Nature Conservation 2018). Split-plot designs include two paired 100-m^2^ plots at the field edge and in the interior (Meyer et al. 2015; Seifert et al. 2014, 2015); three 33-m^2^ plots placed randomly in the interior (Gabriel et al. 2005); two field edge and two field interior plots of each 30 m^2^ (Roschewitz et al. 2005); three plots placed randomly in the interior and one edge plot of each 50 m^2^ (Pinke et al. 2012); two 2000-m^2^ plots in the field interior (Fried et al. 2008); and ten randomly placed 0.1-m^2^ plots (total area only 1 m^2^) (Hanzlik and Gerowitt 2011). Other authors used transects, e.g., one transect at the field edge and one in the interior consisting each of ten 5-m^2^ plots (Clough et al. 2007; Gabriel et al. 2006), one transect at the field edge and one in the interior consisting each of four or five 5-m^2^ plots (Batáry et al. 2012; Solé-Senan et al. 2014), one transect at the field edge and one in the interior consisting each of five 2-m^2^ plots (Krauss et al. 2011), three to ten 10-m^2^ plots in the field interior (depending on field size; Rotchés-Ribalta et al. 2015), and one transect in the field interior of ten 4-m^2^ plots (Petit et al. 2016). The above-mentioned studies used either square or rectangular plots, but oblong relevé plots placed at the field edge are increasingly plausible today, as the largest part of the remaining species pool is restricted to a narrow band along the field edge. In line with this, Bacaro et al. (2015) analyzed a large vegetation dataset consisting of 604 plots within different habitats (among others farmland, grassland, or forests) and found elongated (rectangular) plots to record significantly more species than square plots. This can probably be traced back to an extended perimeter covered by oblong plots and, thus, the chance to include a wider range of environmental and habitat conditions with the associated plant species. The increasingly patchy distribution of arable plant vegetation in pesticide-treated, intensively managed fields may also suggest to use a larger number of small split plots to address vegetation heterogeneity. Such an approach may also account for the finding that the presence and type of adjacent habitats can influence field edge plant diversity by possible spillover effects (Aavik et al. 2008; Nagy et al. 2018; von Arx et al. 2002).

The large variation in available survey methods renders comparison of results difficult. As accurate data on the status and temporal change of the arable plant vegetation at regional, national, and supranational levels is urgently needed for agronomic and conservation purposes, this methodological diversity is highly unsatisfactory. Several authors have attempted to harmonize arable plant survey methods based on experiences gained in earlier studies. Examples are found in Hanzlik and Gerowitt (2016) and Hatcher and Froud-Williams (2017) who suggest to study several small square plots which can be placed randomly or within transects. It has also been proposed to place w-formed transects in the field interior. Species-area curve analysis may help to define a suitable plot size in the arable fields of interest (Pollnac et al. 2009). An example from an intensively farmed region is the study of Mulugeta et al. (2001) in corn and soybean fields of the USA, which predicted that plot sizes between 32 and 185 m^2^, depending on the tillage regime, would be needed to find 75% of the field’s arable plant species pool in the plot. To our knowledge, a systematic comparative study is missing which employs different arable plant survey methods in conventionally managed arable fields, and that could recommend methodological standardization and assess the effectivity of different methods in terms of time expenditure relative to plant detection success.

In this study, we compare six traditional or novel approaches to survey the arable plant vegetation of conventionally managed arable fields with the aim to identify methods that are efficient but also time-economic. We recognize that most of farmland phytodiversity has disappeared from the field interior (Batáry et al. 2017; Clough et al. 2007; Gabriel et al. 2006; Seifert et al. 2014) and that survey methods today have to focus on the narrow field edge strip, which is often less than 2 m wide. We also accounted for the unwillingness of most farmers to allow vegetation relevés in the field interior and thus focused on plots in the edge zone. We thus selected six different survey methods which use oblong plots of different size and placement in the field, (1) a 100-m^2^ plot (50 × 2 m) in the field interior (“Interior”), which also was oblong and mostly served for comparison, (2) an equally sized 100-m^2^ plot at the field edge (“Edge_50”), (3) a 60-m^2^ plot (30 × 2 m, “Edge_30”) at the field edge, (4) a 100-m^2^ oblong plot placed at the field corner (50 × 2 m, “Corner”), (5) a plot area of 20 m^2^ split into four dispersed subplots of 5 m^2^ each (“Subplots”), and (6) an oblong 500-m^2^ plot (500 × 1 m, “Edge_500”) along the field edge. The six approaches differ in plot size (20 to 500 m^2^), plot location in the field (interior vs. edge, corner vs. middle edge), and amount of time needed for survey.

We tested the following hypotheses:

i. Due to the patchy occurrence of the impoverished arable vegetation of today’s intensively managed arable fields, the species detection rate will be highest in oblong 500-m^2^ plots at the field edge and lowest in 100-m^2^ plots in the field interior, where the most intensive management takes place.
ii. Field corner plots are species-richer than field edge plots of similar size.
iii. Beta diversity is lowest among Interior plots due to the universal occurrence of a small set of species well adapted to intensive field management.
iv. Additional species, i.e., taxa not detected with other survey methods, are only found in edge plots and not in the field interior.
v. Splitting plots into subplots increases the number of species recorded per plot area but also increases time expenditure.

## Material and methods

### Study region

The study was conducted in an intensively managed agricultural landscape in the districts of Nienburg (centroid: N 52°36'32.5334'', E 9°6'49.7118'') and Diepholz (N 52°43'41.4940'', E 8°42'4.1629'') in the Pleistocene lowlands of western Lower Saxony (Northwest Germany; Online Resource 6). The districts are part of the natural regions “Ems-Hunte-Geest and Dümmer-Geestniederung” and “Weser-Aller-Flachland” south of the city of Bremen. Most of the farmland is conventionally managed arable land used to produce cereals, maize, and rapeseed for the world market or for use in local biogas plants. The climate of the two districts is temperate-oceanic with mean annual precipitation (2013–2017) of 662.0 mm in Nienburg and 683.6 mm in Diepholz and mean annual temperatures of 10.3 °C (Nienburg) and 10.1 °C (Diepholz) (DWD 2018). The soils (mainly Cambisols, Podzols, Luvisols, and Gleysols) are moderately fertile to fertile and developed from sandy to loamy deposits of the penultimate glaciation (Saalian) or Holocene loess deposits (BGR 2013; LBEG 2015). The farms included in the study were selected by the Chamber of Agriculture of Lower Saxony according to the criteria (i) conventional farming with a relatively high share of cereals, maize, and rapeseed, (ii) more or less even distribution of the farms in the two districts to avoid clumping and spatial autocorrelation, and (iii) willingness of the farmers to participate in the survey.

### Vegetation survey

**Fig. 1:**
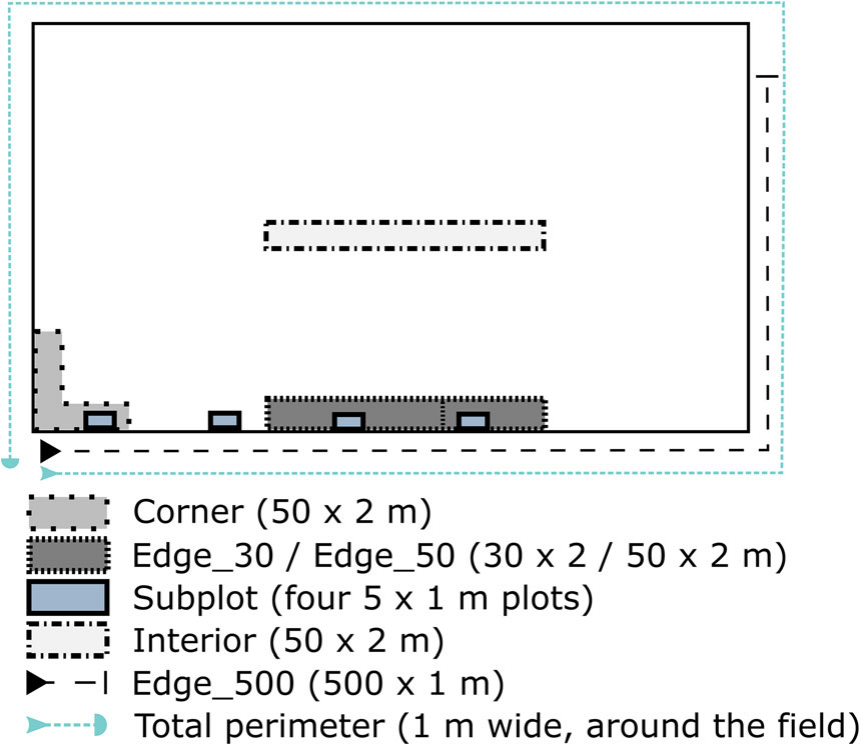
Schematic map displaying the six survey methods that were tested in the study and their location in an arable field (for details see Material and Methods); survey methods: a) 100-m^2^ plots in the field interior (“Interior”; size 50 × 2 m, placed at least 20 m from the edge into the field), b) 60-m^2^ plots at the field edge (“Edge_30”; size 30 × 2 m, along the edge at least 10 m from field corners), c) 100-m^2^ plots at the field edge (“Edge_50”; size 50 × 2 m, along the edge at least 10 m from field corners), d) 100-m^2^ plots at field corners (“Corner”; plots of 50 × 2 m size, placed with two equally sized legs in the field corner), e) four dispersed subplots of 20 m^2^ total size (“Subplots”; each 5 × 1 m, at the field edge at least 10 m distant to each other and to the field corner), and f) linear edge plots of 500 m length and 1 m width (500 m^2^, “Edge_500”)

The vegetation survey with determination of species identity and species richness was carried out from May to July 2017 in 45 fields (ranging in size from 3 to 11 ha) owned by 17 farmers. Fifteen fields had been planted with winter wheat, 15 with maize, and another 15 with winter rapeseed, which are the three dominant crops of the region and are of paramount economic importance for Central European agronomy. In the arable fields, we employed six different approaches to analyze the species richness of the arable plant vegetation at different spatial scales from the level of small plots (5 m^2^) to the field level (see schematic in Fig. 1). Some of the approaches have been used for a long time in vegetation surveys of farmland; others were adopted from more recent vegetation surveys or were introduced by us in response to floristic impoverishment. Since most of the floristic diversity in conventionally managed arable fields is found today in the narrow field edge, we used oblong plots of only 1 to 2 m width to account for the highly heterogeneous distribution of arable plants. We recorded all herbaceous plant species (grasses included; bryophytes excluded) except for juvenile woody plants and crop species. Species names follow Buttler (2018).

In all 45 fields, the following plot and transect types were studied: a) 100-m^2^ plots in the field interior (“Interior”; size 50 × 2 m, placed at least 20 m from the edge into the field), b) 60-m^2^ plots at the field edge (“Edge_30”; size 30 × 2 m, along the edge at least 10 m from field corners), c) 100-m^2^ plots at the field edge (“Edge_50”; size 50 × 2 m, along the edge at least 10 m from field corners), d) 100-m^2^ plots at field corners (“Corner”; plots of 50 × 2 m size, placed with two equally-sized legs in the field corner), e) four dispersed subplots of 20 m^2^ total size (“Subplots”; each 5 × 1 m, at the field edge at least 10 m distant to each other and to the field corner), and f) linear edge plots of 500 m length and 1 m width (500 m^2^, “Edge_500”) along the field edge (consisting of twenty segments of 25 × 1 m each, including at least one field corner) covering 30–70% of the field’s total perimeter. For obtaining an estimate of the field’s overall species pool, we further inspected the 700 to 1500-m-long total perimeter of the field (depending on field size) and counted all herbaceous plant species present along the 1-m-wide margin (line plots of 700–1500 m^2^; “total perimeter”). Based on our experience, we assumed that no additional species are occurring in the field Interior. This species count was used as a reference for the six approaches described above. However, if additional species were observed in a given field in other survey methods, they were added to the total species number.

We used a general plot size of 100 m^2^. In two cases, we also studied smaller plots: approach (b) uses 60 m^2^ in accordance with the plot size and design of the national farmland vegetation monitoring program of Germany (German Federal Agency for Nature Conservation 2018), while approach (e) studies four small plots of 5 m^2^ each, following the sampling scheme of Solé-Senan et al. (2014) and Batáry et al. (2012). The latter approach with several small plots addresses the assumed high within-field variability in arable plant vegetation composition. Field edge plots were either 1 (approaches e and f) or 2 m wide (approaches a to d) and were aligned with the outermost furrow as a plot border. The location of the plots at the field edge and the starting points of the transects were selected by random (see Fig. 1). In each plot, all herbaceous species were listed, and the expenditure of time was noted (all approaches except for b, where the time was not recorded). In approach (e), the total species number was calculated by summing over the four subplots. The location of all plots was determined with GPS. The raw data of the vegetation surveys are compiled in the Online Resource 10 (exclusive crop species, woody seedlings, and few plant individuals which were non-determinable to species level; also excluded from further analysis).

### Statistical analysis

Since most data sets were non-normally distributed, we present median values and use box-whisker plots to visualize species richness data and the time consumed in the different survey methods. This was done for total species richness (all herbaceous non-crop species, including grasses, but without woody plant seedlings), the richness of arable plants sensu stricto according to the definition of Hofmeister and Garve (2006), and the richness of high-nature-value species of arable land (according to the classification of the German Federal Agency for Nature Conservation 2018). High-nature-value species are defined as taxa which characterize through their presence farmland with high conservation value. The conservation value increases with the number of occurring high-nature-value species. We also compared the survey methods for the number of recorded red-listed arable plant species (Red List of Lower Saxony; Garve 2004).

All statistical analyses were conducted with R 3.5.2 software (R Core Team 2018) using the R packages “magrittr” and “tidyverse”, among others (Bache and Wickham 2014; Wickham 2017). To test for spatial autocorrelation among the data from different fields, we calculated Moran’s I (Paradis and Schliep 2018). Since no autocorrelation was detected, all 45 fields were treated as independent data points. To explore the effects of survey methods (explanatory variable) on species richness per plot (response variable), we employed generalized linear mixed-effects models (both with and without negative binomial family; R package “lme4”; Bates et al. 2015) using farmer and crop type as random factors to consider possible individual management effects and crop-dependent diversity patterns. To assess the goodness of the model in terms of the normality and heteroscedasticity of residuals, we inspected the residuals vs. the fitted values and QQ-plots, checked for over-dispersion (Bolker 2019), and calculated the variance explained (R^2^) by fixed effects and by the entire model (Barton 2018; Fox et al. 2011). To test for significant effects of the explanatory variable (survey method) on species richness, we performed likelihood-ratio tests using type II sums of squares as criterion (R package “car”; Fox et al. 2011). Subsequently, a post hoc two-sided Tukey test was applied (R package “multcomp”; Hothorn et al. 2008) to test for significant differences between survey methods. We also tested for differences between survey methods with respect to crop type effects. Due to the rather small sample size per crop (*n* = 15 fields per crop type), we used the Mann-Whitney U test for pairwise comparisons. Since crop type had a large effect on the time consumed in the different survey methods (as exemplified by hardly penetrable rapeseed vs. better accessible wheat fields), mixed-effects models were not applied to explore the effect of survey method on the expenditure of time in the total data set (all crops pooled). As an alternative, we compared the expenditure of time in the different methods for the three crop types separately using the Mann-Whitney U test. We also tested for differences between the survey methods in beta diversity among fields using Jaccard’s similarity index as criterion (R package “vegan”; Oksanen et al. 2019). Significant differences in beta diversity between plots were also tested with the Mann-Whitney U test. In addition, we plotted the species richness found by examining the six survey methods in the 45 fields against the estimated total species number in that field (“total perimeter”) and calculated Spearman’s rank correlation coefficient for the six relations to evaluate the accuracy of the different survey methods in terms of species detection. Finally, species accumulation curves were calculated with the R package “vegan” (method = “random”; permutations = 100; Oksanen et al. 2019) to analyze the influence of the survey method on the increase in species number with plot number or surveyed plot area. Information about model structures and test statistics is presented in Online Resource 9a and 9b.

## Results

### Recorded species richness

**Fig. 2:**
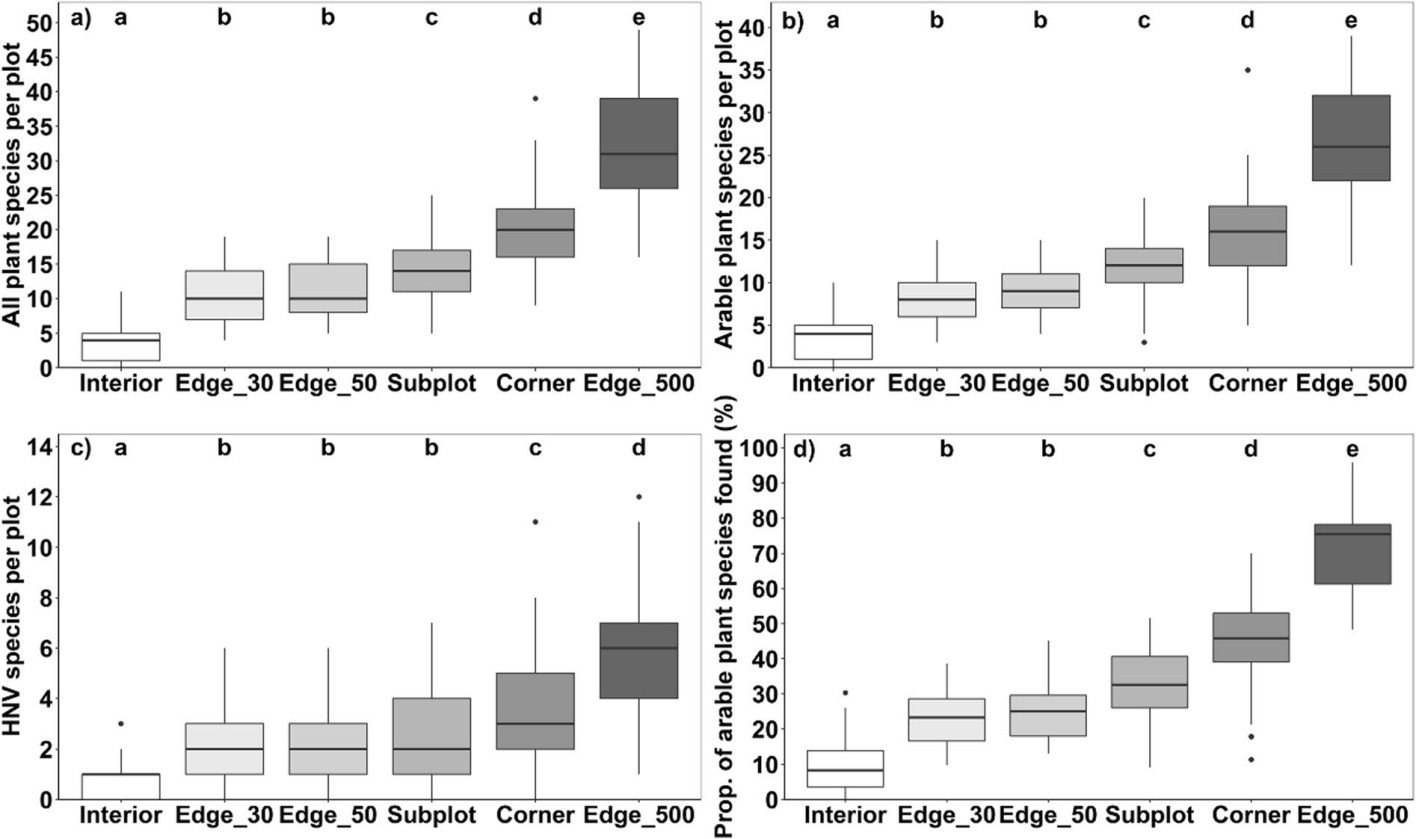
Total number of herbaceous plant species (all plant species, woody seedlings and crops excluded) (**a**), number of arable plant species sensu stricto (as listed by Hofmeister and Garve 2006) (**b**), and number of high-nature-value species (HNV according to the German Federal Agency for Nature Conservation 2018) (**c**) in plots surveyed with six different methods. **(d)** Proportion (in %) of the number of arable plant species sensu stricto present in the field that is found in plots of the six survey methods. All data are averages over wheat, maize, and rapeseed fields. Black lines in boxplots represent medians; *n* = 45 (3 crop types × 15 replicates per crop for each survey method), Tukey’s test, α ≤ 0.05; different small letters indicate significant differences between survey methods methods. For model overview and statistical results see Online Resource 9a and 9b

In the pooled sample of all 45 fields, we observed 197 herbaceous non-crop species (excluding seedlings of woody plants) and 125 arable plant species sensu stricto, when combining the results of all six survey methods (Online Resource 7; see Online Resource 8 for species richness per crop). The estimated total species pool of single wheat, maize, and rapeseed fields in the study region consisted of 45, 40, and 52 herbaceous plant species and 38, 32, and 40 arable plant species, respectively (median values), according to the total perimeter count. The highest total herbaceous species number in the plots was found in the 500 m^2^ Edge_500 plots (median of the three crops: 31 species), followed by the 100-m^2^ Corner plots (20) and the Subplots (14; Fig. 2). Edge_30 and Edge_50 plots (60 and 100 m^2^, respectively) reached very similar, but much lower, species richness (median = 10 in both cases) than the aforementioned survey methods. The lowest median values were recorded in the 100-m^2^ plots in the field Interior (median = 4 species). The same sequence in species richness was also found for the number of arable plant species (according to the classification of Hofmeister and Garve 2006) and for the high-nature-value species of arable land (with exception of the Subplots; Fig. 2). A similar sequence of methods was also detected, when the three crop types are analyzed separately (Online Resource 1). With a few exceptions, the species richness differences between the six tested methods were significant at *p* < 0.05.

In relation to the estimated total species pool of a field (“total perimeter”), the Edge_500 plots yielded the highest proportion of species recorded (median: 71.1% of all herbaceous plants, and 75.6% of all arable plant species; all crops pooled) followed by the Corner plots (median: 44.7 and 45.8%) and the Subplots (31.3 and 32.6%) (Fig. 2 and Online Resource 2). A much lower share of total species number was recorded in the Edge_50, Edge_30, and Interior plots (median: 24.3, 23.3, and 6.3% for all herbaceous plant species and 25.0, 23.3, and 8.3% for the arable plant species). The proportion of high-nature-value species detected in the plots Edge_500, Corner, Subplots, Edge_50, Edge_30, and Interior was 75.0, 46.2, 30.8, 25.0, 22.2, and 6.7%, respectively, of the number found in the total perimeter count (Online Resource 3). The occurrence of red-listed arable plant species (with respect to Lower Saxony; Garve 2004) was very low. In total, we found nine occurrences of five red-listed species (total perimeter count) in eight of the 45 study fields (five rapeseed and three maize fields). In general, only one red-listed species was found per field (except for one field with two red-listed species). Corner and Edge_500 plots showed a slightly higher detection success compared to the other applied field edge survey methods (detection of four red-listed species vs. two or three occurrences), whereas no red-listed species were found in Interior plots. When analyzing the crop types separately, we found 70.5% of all herbaceous plants of the total perimeter count in the Edge_500 plots in wheat fields, 70.8% in maize, and 76.8% in rapeseed fields.

**Fig. 3:**
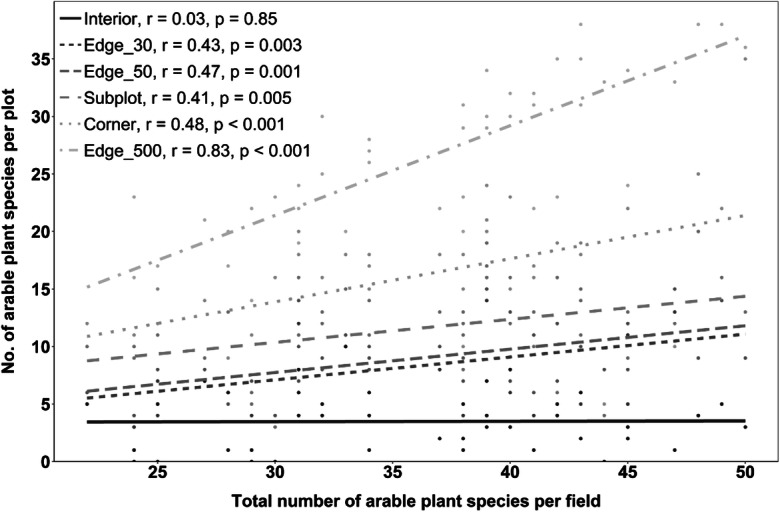
Number of arable plant species found in plots of the six survey methods in relation to total arable plant species number in the respective field; R = Spearman’s rank correlation coefficient with *p* values; n = 45 (per survey method)

The relationship between recorded arable plant species number in a plot and estimated total arable plant species number in the field was strongest for the Edge_500 plots (r = 0.83, *p* < 0.0001), followed by the plot types Corner, Subplots, Edge_50, and Edge_30 (0.48 > r > 0.41; 0.005 > *p* > 0.001, Fig. 3). No correlation was found between the arable plant species richness of Interior plots and total arable plant number per field (r = 0.03). The same pattern was observed for all herbaceous plants instead of arable plant species (Online Resource 4).

### Expenditure of time

**Fig. 4:**
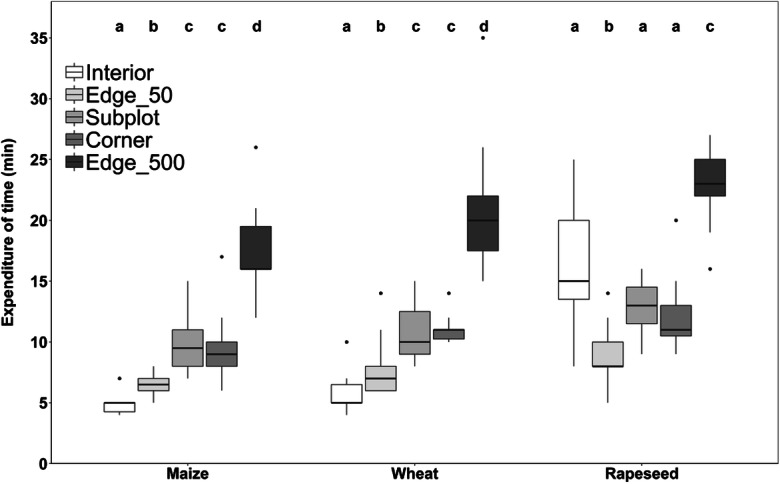
Time required for surveying the plots of the six different survey methods. No expenditure of time measured for Edge_30 plots; black lines in boxplots represent medians; Mann-Whitney U test (pairwise comparisons within crop types using the Wilcoxon rank sum test, α ≤ 0.05); *n* = 15 per crop and survey method (except for each one missing value in a maize Interior plot, maize subplot, maize Edge_50 plot, and wheat Corner plot)

Due to differences in plot size and vegetation density, the six methods differed considerably in the time needed for the survey. Surveying the Edge_500 plots consumed the largest amount of time (median over all crops: 20 min; maize, 16; wheat, 20 min; rapeseed, 23 min; Fig. 4). In maize and wheat, Corner plots and Subplots required only half the time (median: 9–11 min, no significant difference between crop types), followed by Edge_50 plots with even less time (maize: 6.5 min, wheat: 7 min); the fastest surveys were possible in Interior plots (maize and wheat: 5 min; all figures without access to the plot). With 9.5–10 min, the Subplots required more time than the much larger contiguous Edge_30 and Edge_50 plots. Surveying rapeseed plots was in general more time-consuming due to the high crop density; a median value of 15 min was recorded for Interior plots and 23 min for Edge_500 plots.

### Species composition and distribution

Across the whole field sample, the most frequent species were *Elymus repens*, *Chenopodium album*, *Polygonum aviculare*, *Fallopia convolvulus*, *Galium aparine* and *Matricaria chamomilla* (Online Resource 7). Due to the location of the plots in different parts of the field, the six survey methods recorded somewhat different subsets of the total species pool. The species list in Online Resource 7 shows that certain characteristic arable plant species, high-nature-value species, and red-listed species were only recorded with the more labor-intensive methods, notably the Edge_500 plots and the Corner plots. These methods recorded a number of taxa that were not found with the other methods: 34 species were only found in the Edge_500 plots, of which 12 were typical arable plants: *Aethusa cynapium*, *Allium vineale*, *Anchusa arvensis*, *Cardamine hirsuta*, *Glebionis segetum*, *Galinsoga quadriradiata*, *Buglossoides arvensis*, *Matricaria discoidea*, *Ornithopus perpusillus*, *Rorippa palustris*, *Senecio vulgaris*. Four species occurred only in the Corner plots, of which two were typical arable plants (*Anthemis arvensis* and *Anthriscus caucalis*); one typical arable plant species (*Urtica urens*) was recorded only in the Subplots. There were no species which were solely found in Interior plots, Edge_30, and Edge_50 plots. There were also some herbaceous species exclusively appearing in a certain crop, 20 species in maize, 14 in wheat, and 25 in rapeseed (for details, see Online Resource 8). High-nature-value species occurred only very occasionally in Interior plots and always in very small numbers (Online Resource 7). Only five red-listed species were detected with all methods, namely, *Agrostemma githago*, *Anthemis arvensis*, *Buglossoides arvensis*, *Chenopodium hybridum*, and *Odontites vernus*. No threatened species were found in wheat fields and in the interior of rapeseed and maize fields.

**Fig. 5:**
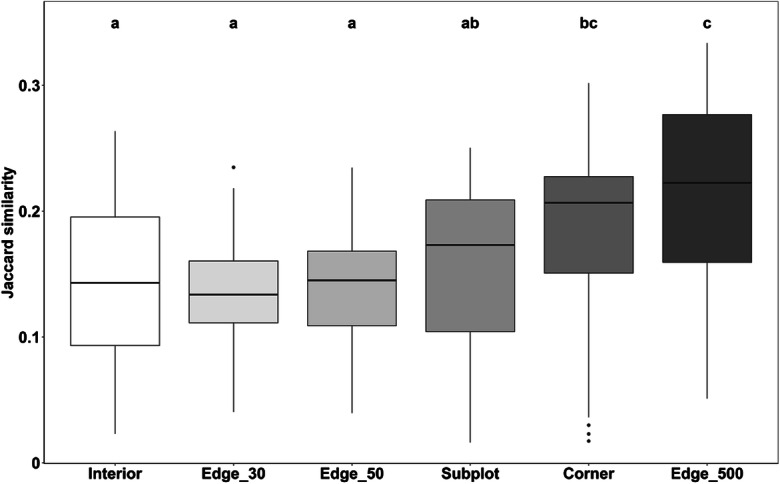
Jaccard similarity index for plots of the six survey methods in the 45 fields. Basis for the calculation were the number of arable plant species sensu stricto according to Hofmeister and Garve (2006). The higher the value, the more similar are species compositions for a given survey method (1 = total similarity). n = 45 for each survey method, except Interior *n* = 40 (5 plots with no species were excluded); different small letters indicate significant differences between survey methods according to a Mann-Whitney U test (α ≤ 0.05)

**Fig. 6:**
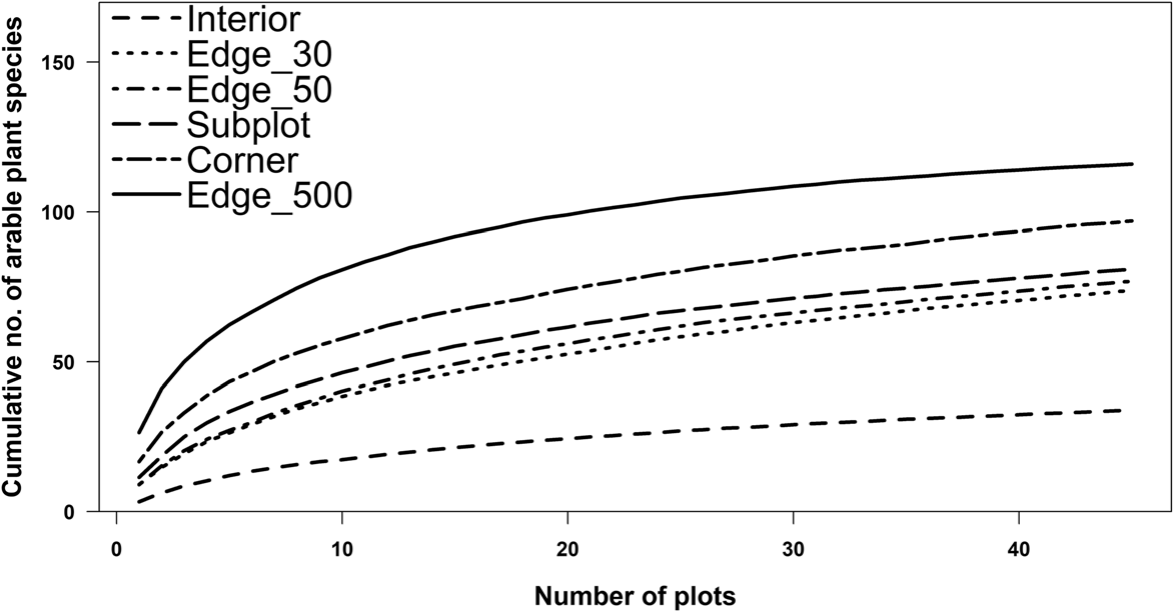
Species accumulation curves for the number of arable plant species for the six survey methods with increasing plot number. Arable plant species sensu stricto according to Hofmeister and Garve (2006); method = “random,” permutations = 100) for different survey methods (see legend); n = 45 for each survey method

Beta diversity, measured with Jaccard’s similarity index for the characteristic arable plant species, was higher for the Interior, Edge_30, and Edge_50 plot surveys (JI: 0.14–0.15; Fig. 5) than for the Edge_500, Corner, and Subplots surveys (JI: 0.17–0.22). Jaccard’s index indicates lower floristic similarity among fields from the data retrieved with the former three methods. Thus, Edge_500 and Corner plots showed highest floristic similarity across the fields. Species accumulation curves for the arable plant species showed a strong increase in species richness for the first 10 plots and the highest initial slope for the Edge_500 plots (Fig. 6). In contrast, the Interior plot curve showed a more continuous increase in species but with very low slope. The other survey methods range between these two extremes. With respect to the twenty 25-m-long subunits investigated in the Edge_500 plots, we found a relatively constant increase in arable plant species richness, until 500 m length was reached (Online Resource 5). Rapeseed showed the steepest, wheat an intermediate, and maize the lowest slope of the species-plot number curve. In none of the crop types, species richness saturated at 500-m plot length, as shown by comparison with the total perimeter count.

## Discussion

Our systematic comparison of six vegetation survey methods demonstrates high within-field heterogeneity in the arable plant vegetation of conventionally managed farmland. About 90% of arable plant species richness that has survived agricultural intensification was found in the 1-m-wide marginal strip of the fields (Fig. 2d). This was similar in the three crop types despite contrasting stand structures, phenology, and management regimes. This suggests that the main factors that drove the bulk of arable plant species out of the field interior are herbicide use and lower light penetration to the ground in the highly fertilized, dense crop stands, in conjunction with the tillage regime as the most influential environmental factors of conventional agriculture. This corroborates the findings of various studies that most of arable plant diversity today is restricted to the edge zone of arable fields (Albrecht et al. 2016; Batáry et al. 2017; Romero et al. 2008; Seifert et al. 2014, 2015). Thus, future arable plant monitoring should focus on field edge areas of 1 to 2 m width.

In line with hypothesis (iv), we did not find a single plant species in the Interior plots of the 45 fields that would have been missed, when exclusively investigating the edge zone. Only a few generalist arable plant species were found more frequently in Interior plots, mainly *Viola arvensis* (in 23 of 45 Interior plots), *Polygonum aviculare* (17), *Chenopodium album* (13), *Matricaria chamomilla* (13), *Stellaria media* (13), and *Fallopia convolvulus* (12; Online Resource 7). In addition, there was no correlation between observed arable plant richness in the Interior plots and total arable plant diversity in the field (r = 0.03; Fig. 3). Only 37 herbaceous plant species in total were recorded in the Interior of the 45 fields, 34 of which were arable plants sensu stricto. Decades of intense management have not only impoverished the actual arable plant vegetation in the field but also the seed bank and forced a large proportion of formerly widespread arable plant taxa to seek refuge in the edge zone or face extinction (Aavik et al. 2008; Andreasen et al. 2018; Meyer et al. 2013, 2015; von Arx et al. 2002). Directly neighboring, extensively managed habitats may nowadays function as source habitats for some plants, which spill over in the intensively managed field and increase plant diversity (Aavik et al. 2008; Nagy et al. 2018; von Arx et al. 2002). Before agricultural intensification, arable plant diversity was with 24 species per 100 m^2^ (median) six times higher in the field interior than it is today, explaining why earlier vegetation surveys focused on these areas (Meyer et al. 2013).

Floristic impoverishment justifies the use of strip-like plots of 2 or better 1 m width along the field edge without losing relevant information. Which of the five tested oblong plots at the field edge is preferable depends on the purpose of study and available time. Rapid surveys in over-regional or national farmland biodiversity monitoring schemes may rely on the 60-m^2^ Edge_30 plots, as used by the German Federal Agency for Nature Conservation (2018), which were surveyed by us in typically 6–7 min, but we recorded with this survey method only 23.3% of the total species number and also of the arable plant species pool (median; Fig. 2 and Online Resource 2). The detection success is only insignificantly higher in Edge_50 plots (100 m^2^), while the time needed is probably only slightly higher (median Edge_50 plots across all crops: 7 min). Therefore, it is mainly a question of standardization, whether 60- or 100-m^2^ plots at the field edge are investigated. Given that further impoverishment in farmland biodiversity is likely to happen, we would recommend to prefer Edge_50 over Edge_30 plots. Our results further suggest that oblong plots located at field corners contain a larger fraction of the field’s species pool than plots in the central part of the margin zone (Fig. 2), irrespective of crop species, which supports hypotheses (i) and (ii). This is certainly a consequence of typically less intensive herbicide use and fertilization at field corners, where tractors are turning, disturbance is higher, and crop seed density is often reduced. Corner plots have the additional advantage that they may be more easily accessible than oblong plots along hedges or grass strips in the middle of the edge. The greatest advantage of oblong 100-m^2^ Corner plots is their reasonable detection success, which is nearly twice as high (45 vs. 25%; Fig. 2) than that of similarly sized Edge_50 plots in the central part of the margin zone, while the expenditure of time is only ca. 64% higher (median 11 vs. 7 min across all crops). As postulated in hypothesis (v), an interesting alternative can be the Subplots approach, which has a similar time requirement as a single Corner plot (12 min) but has the potential to detect a relatively large number of species (32.6% of the field’s arable plant species pool) despite its small cumulated plot size (20 m^2^; Figs. 2 and 4). The effectiveness of this method is explained by the relatively low similarity in species composition in contemporary arable plant communities at the landscape scale, as is displayed in the low Jaccard indices and the relatively steep increase of the species-area curve at low plot numbers (Figs. 5 and 6). However, since the detection success is less than that of Corner plots, while the time needed is similar, the Corner plot approach seems to be more attractive. In addition, the Subplots method requires that the Subplots are placed either strictly by random or at fixed distances to guarantee comparability and to avoid cherry picking in terms of plant diversity along the field edge. If the Subplots approach is adopted, we would suggest placing at least two of the Subplots at the corner to increase the species richness recorded.

Clearly the highest detection success is achieved by the Edge_500 method, which investigates a five times larger plot area (500 m^2^) than all other methods. In smaller fields, this area accounts for more than 50% of the entire field edge area. With a median detection success of 75.6% (arable plant species; Fig. 2), the vegetation of the field is sufficiently well represented in this type of relevé to serve the goals of phytodiversity assessments and long-term monitoring schemes. High-nature-value species, if they occur, will mostly be detected, which is often not the case in the other approaches with less than 50 or even 25% detection success (Online Resource 3). For the Edge_500 approach, we found in the 45 - field sample a highly significant correlation between the number of arable plant species detected in these plots and the total number of arable plant species present in the field (r = 0.83, *p* < 0.0001; Fig. 3). The method is clearly more time-consuming than others (~20 min per plot across all crops) and perhaps not the choice for landscape-level surveys, where the focus is on a large number of plots. Yet, with only 9 min spent additionally (compared to Corner plots), about a quarter of the field’s species pool is additionally recorded (Fig. 2). Moreover, the time needed to access fields and plots (which is not included in our figures) is often much more relevant than the time used for taking the relevé itself. This may convince researchers to shift from smaller plots to the 500-m^2^ plot method, as the gain in additional information is considerable. For vegetation surveys in managed temperate grasslands, Ruff et al. (2013) similarly recommend large oblong plots as the most effective method. Bacaro et al. (2015) also found significantly higher species richness in rectangular (oblong) plots compared to square plots, but they state that plot alignment across possible environmental gradients may influence the species detection success. If the monitoring focus is on red-listed arable plant species, the total perimeter count should be considered. With respect to this species group, even the Edge_500 plots showed only a slightly higher detection success compared to the other field edge survey methods.

By focusing in arable plant surveys on the edge zone, greater mixing of arable plants with taxa from adjacent habitats is to be expected and is frequently reflected in the relevés. This is a clear disadvantage of linear plot surveys along field edges compared to the historical approach of square plots in the field interior. We therefore recommend considering characteristic arable plant species (as listed by Hofmeister and Garve 2006) in the relevé data in addition to the total herbaceous plant species list.

How much time is needed for a vegetation survey depends on crop density and thus season and crop species (Fig. 4). This is clearly demonstrated by rapeseed which generally requires more survey time due to its dense stand shortly before harvest. Surveys earlier in the year, when crop height is lower, may not be a good alternative, because many species are more difficult to identify and some may even be overlooked.

Unexpected is our result that floristic similarity is higher at field edges than in the Interior across the sample of 45 fields (Fig. 5) which contradicts hypothesis (iii). Most vegetation surveys in intensively managed farmland noted a trend toward increasing homogenization of the arable plant vegetation in recent decades (Hanzlik and Gerowitt 2016; Meyer et al. 2013, 2015; Seifert et al. 2015), as phytodiversity has decreased greatly and many formerly rare, specialist taxa have disappeared entirely from the cultural landscape of large parts of Central Europe (Leuschner and Ellenberg 2017). Since the environment is most stressful to plants in the field interior, this suggests that a largely uniform basic set of highly stress- and herbicide-tolerant species should have survived, which is similar in all intensively used fields. We thus expected less between-field variation in community composition on a larger spatial scale (Hanzlik and Gerowitt 2016). In fact, there are very few widespread stress-tolerant species, such as *Viola arvensis*, *Polygonum aviculare*, *Chenopodium album*, *Matricaria chamomilla*, *Stellaria media*, and *Fallopia convolvulus*, which were found in Interior plots more frequently. However, due to the impoverishment of the field interior community, the absence of two or three of the common species greatly increases floristic dissimilarity between fields. Thus, the lower beta diversity of the field edge communities as compared to the Interior plots can be explained by the much lower overall diversity of the latter. In addition, some of the common arable plant species such as *Echinochloa crus-galli*, *Apera spica-venti*, *Myosotis arvensis*, or *Sisymbrium officinale* seem to have preferences in terms of crop species (Online Resource 8), edaphic conditions, field management (pest control, tillage, and fertilization regime), and field neighborhood (Albrecht et al. 2016; Fried et al. 2008; Hanzlik and Gerowitt 2016; Lososová et al. 2004; Meyer et al. 2013; Pinke et al. 2012; Seifert et al. 2014).

Beta diversity, i.e. within- and between-field variation, is a major component of phytodiversity in the studied arable field complex. This is shown by the large interior-edge gradient in plot-level diversity and is also displayed by the species richness increase by 25% from the Edge_500 plot to the entire perimeter count (Fig. 2). Rising species numbers beyond a plot size of 500 m^2^ were also found by Mulugeta et al. (2001) in maize and soybean fields. Inspection of the species-area (plot number) curves further shows that regional variation is also playing a significant role for the landscape-level diversity (gamma diversity) of the region’s arable plant vegetation (Fig. 6). All curves, in particular those of the field edge communities, tended to increase beyond 45 fields, probably reflecting differences in edaphic, climatic, and agronomic conditions in the study region. The different components of phytodiversity need consideration in biodiversity assessment and monitoring schemes.

## Conclusions

This comparison of six approaches to survey arable plant vegetation showed large differences with respect to the proportion of detected species and the expenditure of time. The recent impoverishment of arable vegetation has the consequence that the former quadratic relevé plots should be replaced by oblong strip-like plots at the field edge, preferably across a field corner. Except for studies where large plot numbers have to be surveyed in short time, we recommend 500-m-long and 1-m-wide linear plots at the edge, which include a field corner. An experienced botanist will need no more than about 20 min for the collection of presence/absence data in the 500-m^2^ plot with a high chance to record 75% or more of the field’s overall arable plant diversity. When this is not possible due to time constraints, oblong 100-m^2^ plots stretching over a corner are also a promising option. Such plots should be introduced in addition to a subset of older plots that are surveyed for continuity of methods. Our conclusions about the efficiency of the examined vegetation survey methods should be tested in future studies for additional crop species, in other management systems (e.g., organic farming), and in additional regions such as the Mediterranean, where more species-rich farmland is still present. In addition, the influence of landscape heterogeneity and composition and the role of adjacent habitats on the arable plant diversity should be studied in more detail. Finally, more attention should be paid to between-field differences in segetal community composition and their causes. In the light of the large within- and between-field variation in species richness and the resulting poor comparability of relevé data, monitoring agencies should take initiatives to harmonize arable plant vegetation survey methods at national and international levels.

## Electronic supplementary material

ESM 1-6 Online Resource 1 Total number of herbaceous species observed in the different crop types.
Online Resource 2 Proportion of the herbaceous species number found in the plots of the different survey methods relative to the field’s total species pool (total perimeter count; all herbaceous species considered).
Online Resource 3 Proportion of the number of high-nature-value (HNV) species found in the plots of the different survey methods relative to the field’s total HNV species pool (total perimeter count; all herbaceous species considered).
Online Resource 4 Number of all herbaceous plant species found in plots of the six survey methods in relation to the field’s total herbaceous species number.
Online Resource 5 Cumulative number of arable plant species sensu stricto in the 20 sections of the Edge_500 plots.
Online Resource 6 Map of the study region (PDF 539 kb)

ESM 7 Online Resource 7 Frequency of all herbaceous species in the relevés done with the different survey methods (all crop species pooled) (CSV 8.75 kb)

ESM 8 Online Resource 8 Frequency of herbaceous species in the relevés for the seven crop species (CSV 7.32 kb)

ESM 9 Online Resource 9a and 9b Overview of used models and statistical results (XLSX 26.2 kb)

ESM 10 Online Resource 10 Raw data (XLSX 216 kb)
