# Supplementary material for: Surveying the arable plant diversity of conventionally managed farmland: a comparison of methods
Source: Environ Monit Assess. 2020 Jan 7;192(2):98. doi: 10.1007/s10661-019-8042-7 (PMC8076134; doi:10.1007/s10661-019-8042-7)
Supplement: Supplementary file 1 — Online Resource 1 Total number of herbaceous species observed in the different crop types. Online Resource 2 Proportion of the herbaceous species number found in the plots of the different survey methods relative to the field’s total species pool (total perimeter count; all herbaceous species considered). Online Resource 3 Proportion of the number of high-nature-value (HNV) species found in the plots of the different survey methods relative to the field’s total HNV species pool (total perimeter count; all herbaceous species considered). Online Resource 4 Number of all herbaceous plant species found in plots of the six survey methods in relation to the field’s total herbaceous species number. Online Resource 5 Cumulative number of arable plant species sensu stricto in the 20 sections of the Edge_500 plots. Online Resource 6 Map of the study region (PDF 539 kb) [file 10661_2019_8042_MOESM1_ESM.pdf]

Electronic Supplementary Material to the paper

Surveying the arable plant diversity of conventionally managed farmland: a comparison of methods. *Environmental Monitoring and Assessment.* Alexander Wietzke[1] Christoph Leuschner.

[1]Plant Ecology and Ecosystems Research, University of Goettingen; E-mail address: alexander.wietzke@biologie.uni-goettingen.de

Online Resource 1 Total number of herbaceous species observed in the different crop types

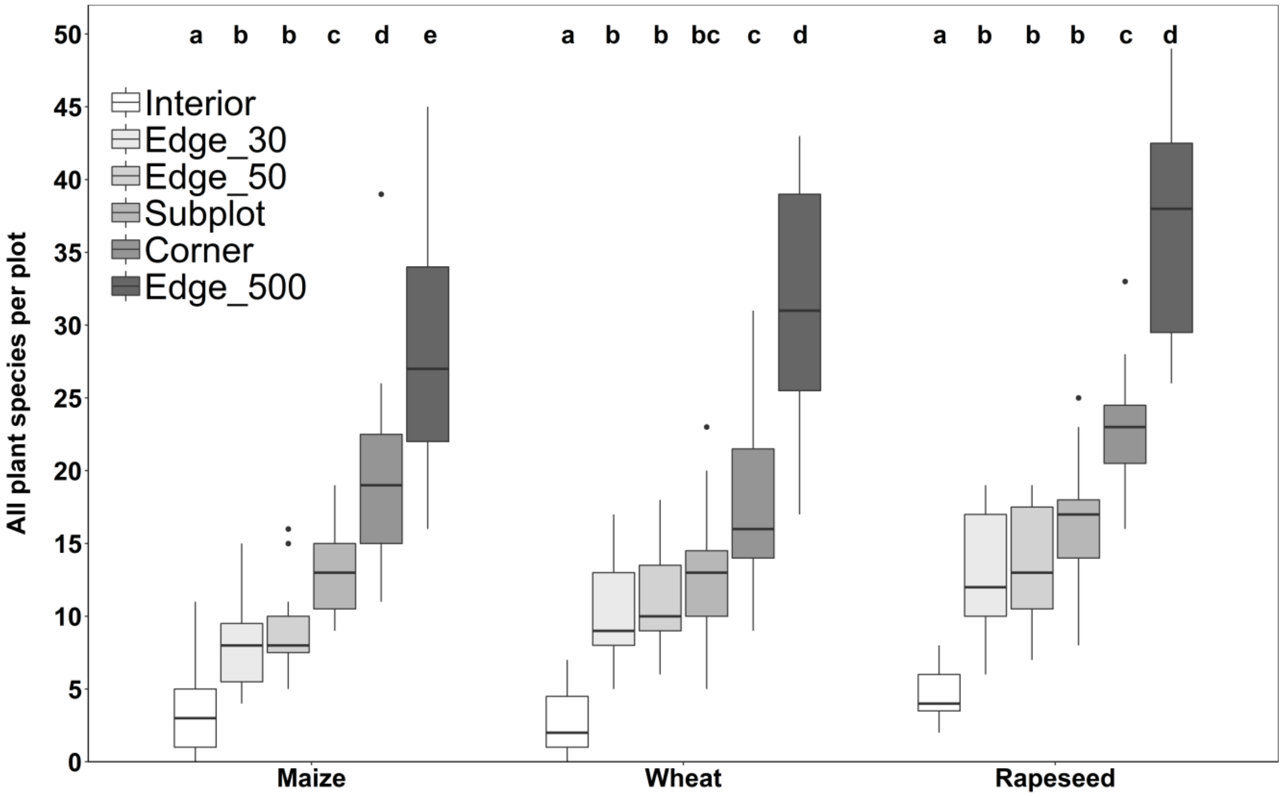

Online Resource 1 Total number of herbaceous species observed in the different crop types (winter wheat, winter rapeseed, maize); species number exclusive crop species and woody seedlings; survey methods in different colors (see legend): Interior (50 x 2 m), Edge_30 (30 x 2 m), Edge_50 (50 x 2 m), Subplots (four plots of 5 x 1 m), Corner (50 x 2 m) and Edge_500 (500 x 1 m); black lines in boxplots represent medians; Mann-Whitney-U-Test (pairwise comparisons within crop types using Wilcoxon rank sum test, $\alpha \leq 0.05$); n = 15 per crop and survey method; different small letters indicate significant differences between survey methods within crop types; model overview and statistical results see Online Resource 9

Electronic Supplementary Material to the paper
Surveying the arable plant diversity of conventionally managed farmland: a comparison of methods. *Environmental Monitoring and Assessment.* Alexander Wietzke[1] Christoph Leuschner.

[1]Plant Ecology and Ecosystems Research, University of Goettingen; E-mail address: alexander.wietzke@biologie.uni-goettingen.de

Online Resource 2 Proportion of the herbaceous species number found in the plots of the different survey methods relative to the field's total species pool (total perimeter count; all herbaceous species considered)

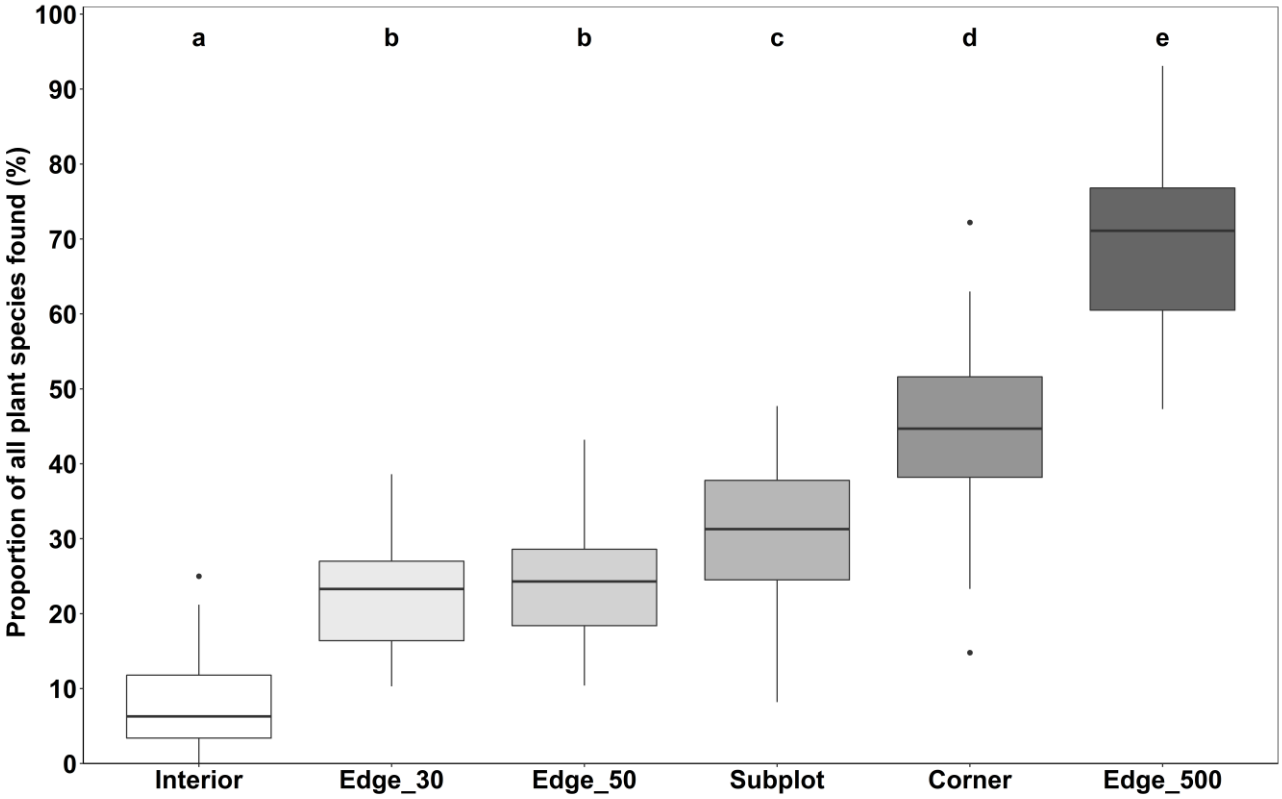

Online Resource 2 Proportion of the species number found in the plots (%) of the different survey methods relative to the field's total species pool (total perimeter count; all herbaceous species considered, crops and woody seedlings excluded; winter wheat, winter rapeseed and maize were pooled); Interior (50 x 2 m), Edge_30 (30 x 2 m), Edge_50 (50 x 2 m), Subplots (four plots of 5 x 1 m), Corner (50 x 2 m) and Edge_500 (500 x 1 m); black lines in boxplots represent medians; n = 45 (per survey method), Tukey's test α ≤ 0.05; different small letters indicate significant differences between survey methods; model overview and statistical results see Online Resource 9

Electronic Supplementary Material to the paper

Surveying the arable plant diversity of conventionally managed farmland: a comparison of methods. *Environmental Monitoring and Assessment.* Alexander Wietzke[1] Christoph Leuschner.

[1]Plant Ecology and Ecosystems Research, University of Goettingen; E-mail address: alexander.wietzke@biologie.uni-goettingen.de

Online Resource 3 Proportion of the number of high-nature-value (HNV) species found in the plots of the different survey methods relative to the field's total HNV species pool (total perimeter count; all herbaceous species considered)

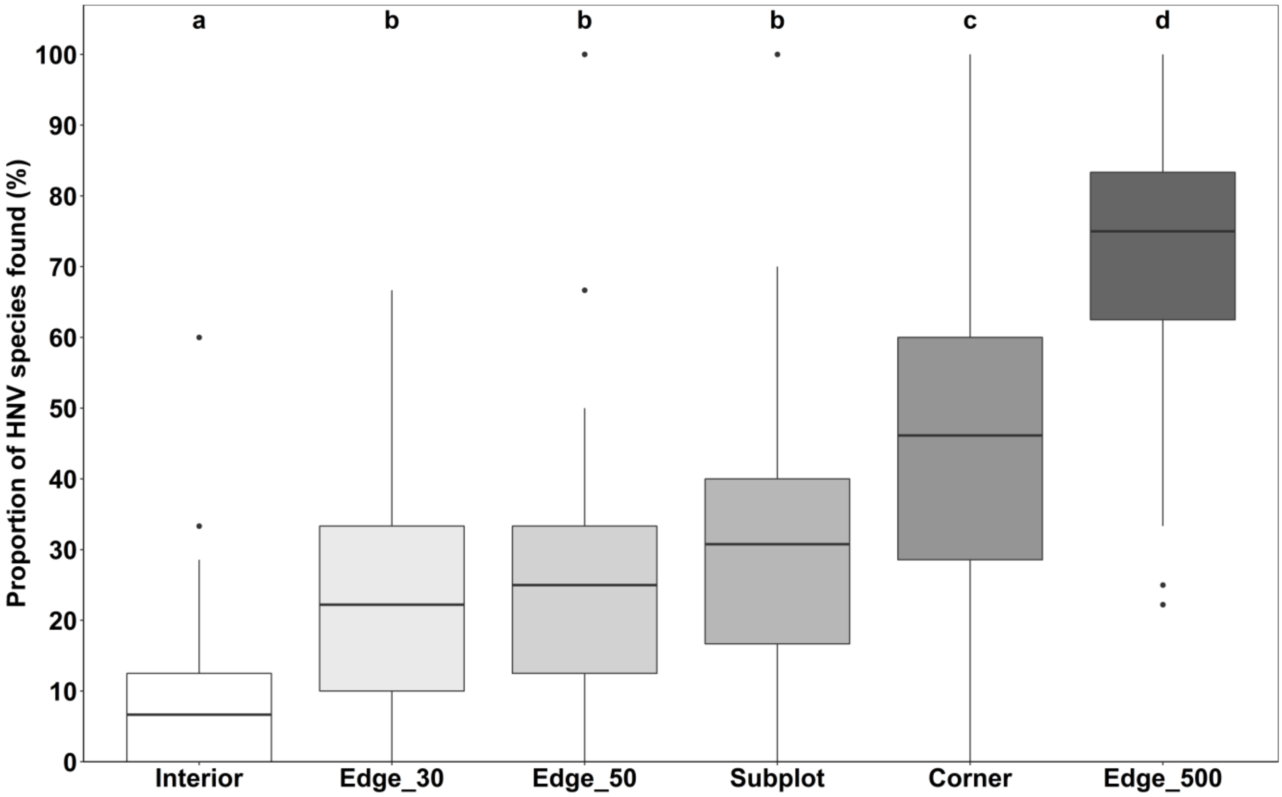

Online Resource 3 Proportion of the number of high-nature-value (HNV; according to German Federal Agency for Nature Conservation, 2018) species (%) found in the plots of the different survey methods relative to the field's total high-nature-value (HNV) species pool (total perimeter count; all herbaceous species considered, crops and woody seedlings excluded; winter wheat, winter rapeseed and maize were pooled); Interior (50 x 2 m), Edge_30 (30 x 2 m), Edge_50 (50 x 2 m), Subplots (four plots of 5 x 1 m), Corner (50 x 2 m) and Edge_500 (500 x 1 m); black lines in boxplots represent medians; n = 45 (per survey method), Tukey's test α ≤ 0.05; different small letters indicate significant differences between survey methods; model overview and statistical results see Online Resource 9

References

German Federal Agency for Nature Conservation (2018). High-nature-value farmland, Germany. https://www.bfn.de/themen/monitoring/monitoring-von-landwirtschaftsflaechen-mit-hohem-naturwert.html. Accessed 19 October 2018.

Electronic Supplementary Material to the paper
Surveying the arable plant diversity of conventionally managed farmland: a comparison of methods. *Environmental Monitoring and Assessment.* Alexander Wietzke[1] Christoph Leuschner.

[1]Plant Ecology and Ecosystems Research, University of Goettingen; E-mail address: alexander.wietzke@biologie.uni-goettingen.de

Online Resource 4 Number of all herbaceous plant species found in plots of the six survey methods in relation to the field's total herbaceous species number

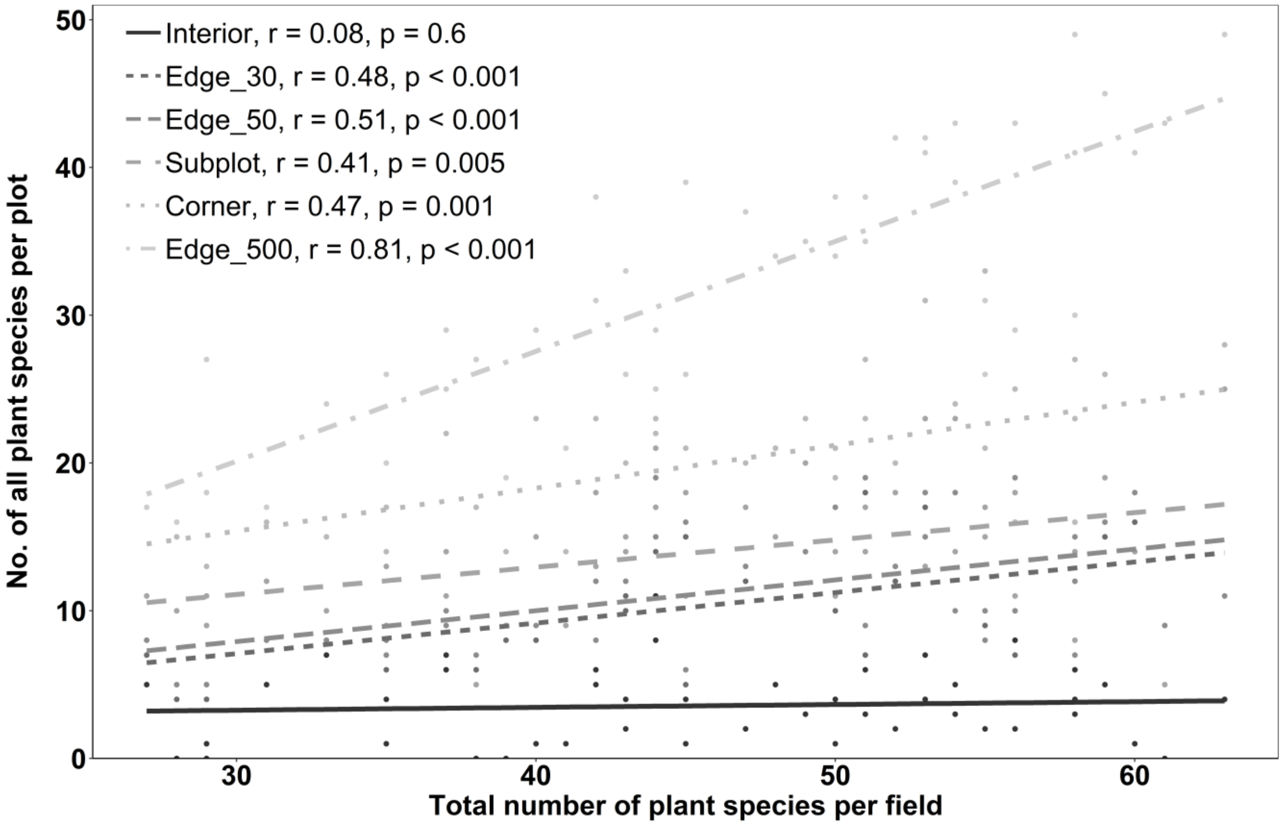

Online Resource 4 Number of all herbaceous plant species found in plots of the six survey methods in relation to the field's total herbaceous species number (crops and woody seedlings excluded); survey method (see legend): Interior (50 x 2 m), Edge_30 (30 x 2 m), Edge_50 (50 x 2 m), Subplots (four plots of 5 x 1 m), Corner (50 x 2 m) and Edge_500 (500 x 1 m); R = Spearman's rank correlation coefficient with p-values; n = 45 (per survey method)

Electronic Supplementary Material to the paper
Surveying the arable plant diversity of conventionally managed farmland: a comparison of methods. *Environmental Monitoring and Assessment.* Alexander Wietzke[1] Christoph Leuschner.

[1]Plant Ecology and Ecosystems Research, University of Goettingen; E-mail address: alexander.wietzke@biologie.uni-goettingen.de

Online Resource 5 Cumulative number of arable plant species sensu stricto in the 20 sections of the Edge_500 plots

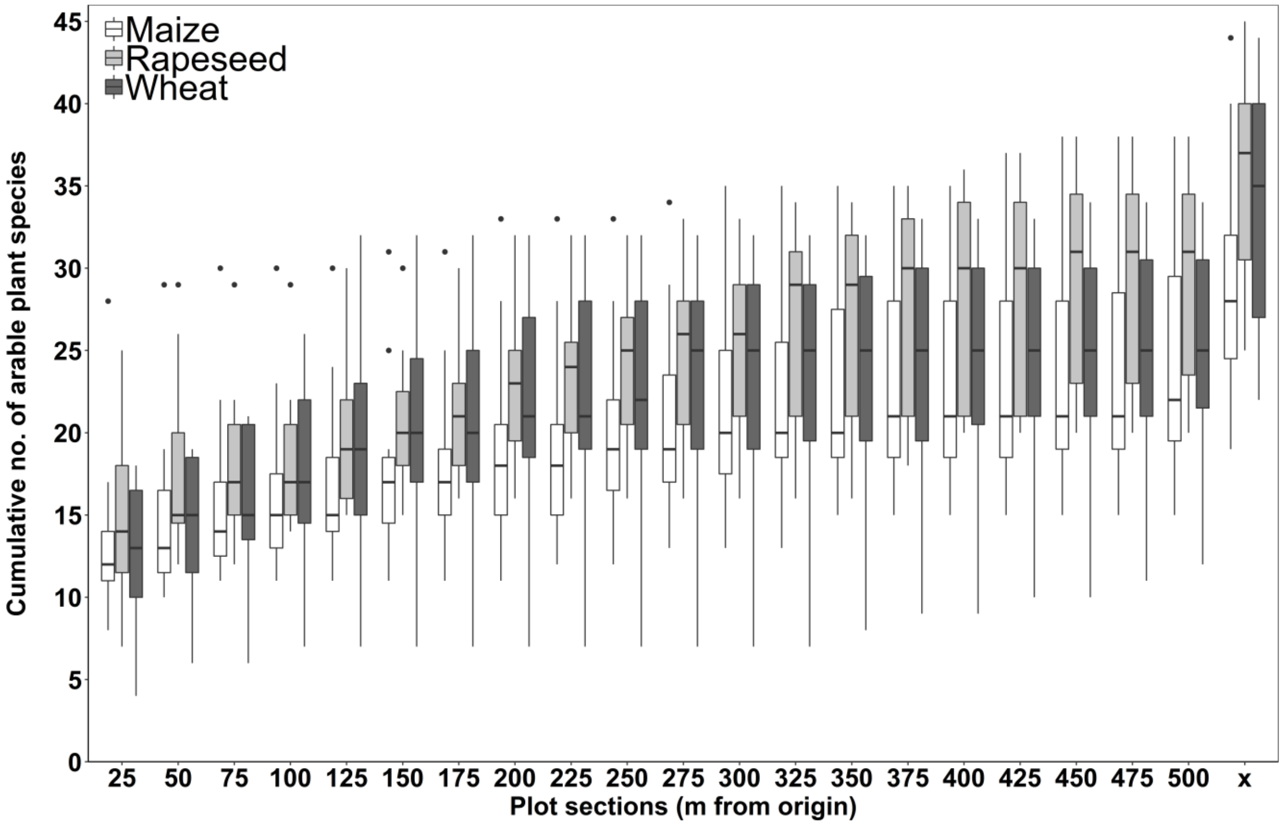

Online Resource 5 Cumulative number of arable plant species sensu stricto (Hofmeister & Garve, 2006) in the 20 sections (each of 25 m length) of the Edge_500 plots (size: 500 x 1 m) in wheat, rapeseed and maize fields (see legend); "x" = additional species found subsequent to the 500 m plots (field perimeter ranged between 700 and 1500 m); n = 45; black lines in boxplots represent medians

References

Hofmeister, H., & Garve, E. (2006). *Lebensraum Acker* (Reprint der 2. neubearbeiteten Auflage). Remagen: Verlag Kessel.

Electronic Supplementary Material to the paper
Surveying the arable plant diversity of conventionally managed farmland: a comparison of methods. *Environmental Monitoring and Assessment.* Alexander Wietzke[1] Christoph Leuschner.
[1]Plant Ecology and Ecosystems Research, University of Goettingen; E-mail address: alexander.wietzke@biologie.uni-goettingen.de

Online Resource 6 Map of the study region

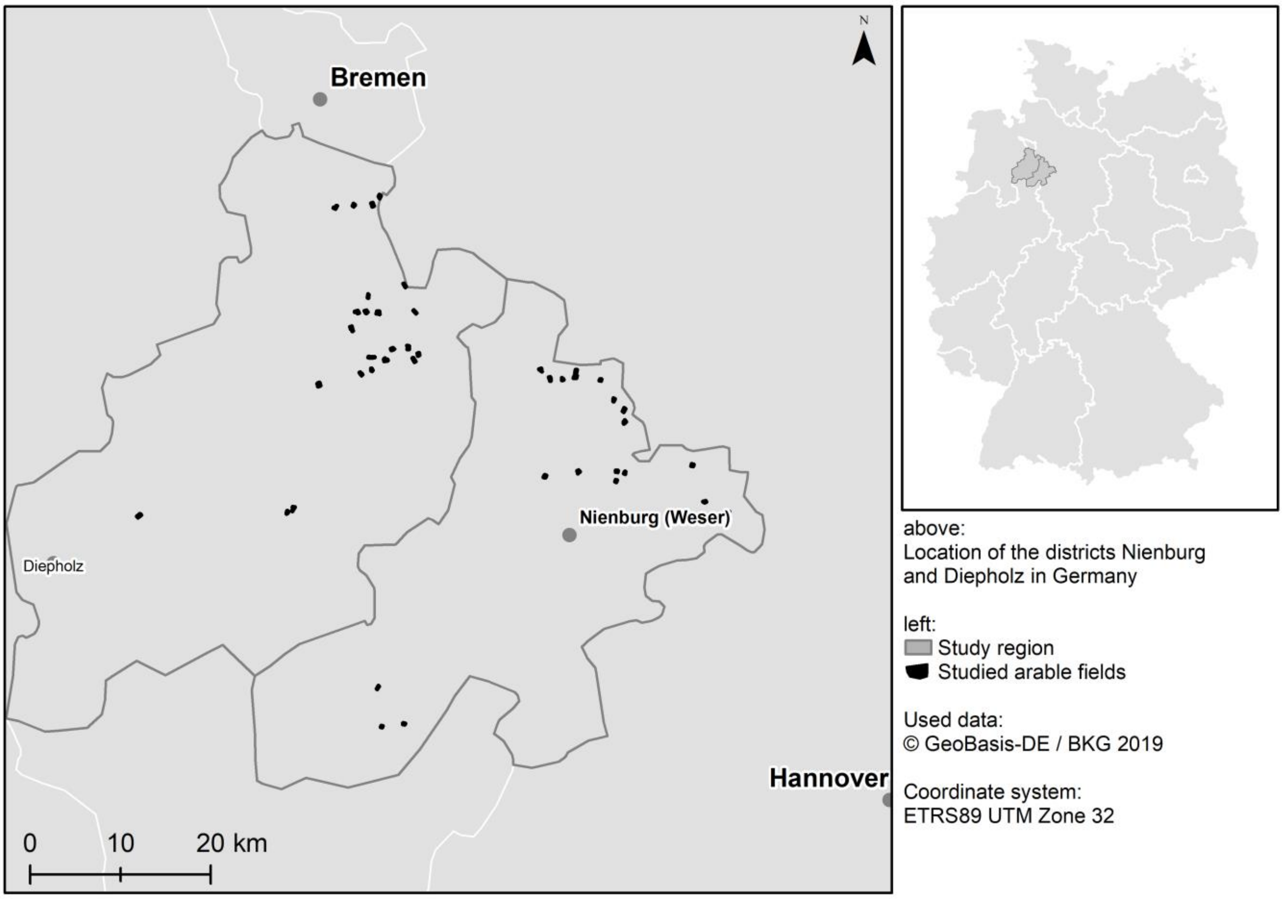

Online Resource 6 Map of the study region (the two districts of Nienburg and Diepholz in Lower Saxony, Northwest Germany)
